# Inflammation in acute heart failure

**DOI:** 10.3389/fcvm.2023.1235178

**Published:** 2023-11-17

**Authors:** Manuel Garofalo, Rossana Corso, Daniela Tomasoni, Marianna Adamo, Carlo M. Lombardi, Riccardo M. Inciardi, Cristina Gussago, Carlo Di Mario, Marco Metra, Matteo Pagnesi

**Affiliations:** ^1^ Department of Cardiology, Careggi University Hospital, Florence, Italy; ^2^ Department of Internal Medicine, ASST Sette Laghi, Varese, Italy; ^3^ Cardiology, ASST Spedali Civili and Department of Medical and Surgical Specialties, Radiological Science and Public Health, University of Brescia, Brescia, Italy

**Keywords:** heart failure, acute heart failure (AHF), inflammation, systemic inflammation, anti-inflammatory therapy

## Abstract

Acute heart failure (AHF) represents a common clinical scenario that requires prompt evaluation and therapy and that is characterized by a high risk of mortality or subsequent rehospitalizations. The pathophysiology leading to AHF decompensation is still not fully understood. Significant activation of inflammatory pathways has been identified in patients with AHF, particularly in its most severe forms, and it has been hypothesized that systemic inflammation has a role in AHF pathogenesis. Several inflammatory mediators and cytokines, such as high sensitivity C-reactive protein, tumor necrosis factor-α, interleukin-6, interleukin-1, soluble suppression of tumorigenicity 2 and galectin-3, have been shown to play a role in the pathogenesis, development and worsening of this condition with an independent prediction of adverse outcomes. This manuscript reviews the prevalence and prognostic value of systemic inflammation in AHF, as well as the potential role of anti-inflammatory therapies, focusing on available evidence from clinical trials and ongoing studies.

## Introduction

Acute heart failure (AHF) encompasses a broad spectrum of disease states and is characterized by heterogeneous clinical presentations (1–4). It refers to the onset of symptoms and/or signs of heart failure (HF) leading to an unplanned hospital admission and requiring urgent evaluation and therapy (5). Four major clinical presentations can be described with possible overlaps: acute decompensated heart failure (ADHF), acute pulmonary oedema, isolated right ventricular failure and cardiogenic shock (6). Finally, one-third of AHF presents as new-onset (*de novo*) HF (7). Although AHF is a common disease, with HF-related hospitalizations representing 1%–2% of all Emergency Department (ED) admissions in Europe (8), the pathophysiologic mechanisms underlying acute HF decompensations remain partially unexplained and the management unsatisfactory (9). AHF remains associated with increased mortality both in-hospital and to a larger extent post-discharge (10). The risk of death or rehospitalization is maximal in the few weeks after discharge, the so-called vulnerable phase, and decreases exponentially thereafter though remaining increased compared to that of the patients who remain ambulatory (11–13). In-hospital mortality ranges from 4% to 10% with rates of death or readmission higher than 45% after one year from hospital discharge (5). No evidence-based therapy is able to determine a substantial prognostic improvement during AHF hospitalization (2), although recent trials have demonstrated the benefit of early implementation of guideline-directed medical therapies on outcomes after discharge (14–17).

Systemic inflammation has been frequently identified in patients with both acute and chronic HF, with significantly enhanced neurohormonal and inflammatory activation especially in AHF (9, 18–20), but without a definitive pathophysiological link between inflammation and AHF development (21, 22). There is evidence that inflammation in ADHF may contribute to changes in vascular resistance, fluid redistribution and pulmonary congestion, diastolic dysfunction and systemic hypoperfusion (23, 24). Inflammatory activation also promotes a proapoptotic and prothrombotic milieu (25).

Several inflammatory mediators and cytokines are involved in AHF pathophysiology including high sensitivity C-reactive protein (hsCRP), tumor necrosis factor-alpha (TNF-α), interleukin-6 (IL-6), interleukin-1 (IL-1), soluble suppression of tumorigenesis-2 (sST2) and galectin-3. They have been associated to disease development and progression, representing independent predictors of poor mid-term and long-term outcomes in patients with recent ADHF (26–29). Unfortunately, it has been challenging to translate these prognostic findings into “prognostically-relevant” therapies (30). In fact, the goal of using anti-inflammatory drugs as disease-modifying therapy in AHF has not yet been achieved, with conflicting results from the most important clinical trials conducted in this direction, likely reflecting our poor understanding of the inflammatory networks underlying the heterogenous HF syndrome (31).

In our review, we discuss the role of inflammatory biomarkers in AHF, their prognostic value and the potential utility of targeting precise phenotypes to improve outcomes. We also summarize the results of most important clinical trials of therapies targeting inflammatory processes in the AHF setting, and explore future directions in this field.

## Prevalence and pathophysiology of inflammation in AHF

Numerous studies have confirmed the presence of a sterile inflammation state (without concomitant infections) in ADHF, with high levels of several inflammatory cytokines at hospital admission detected in some patients with AHF. The intensity of systemic inflammation, as identified by different biomarkers, varies in different studies and according to different AHF presentation.

In AHF, the inflammatory response may arise both from antigenic stimulation during infection (e.g., viral myocarditis) or as a result of hemodynamic stress (32, 33). Systemic congestion and/or peripheral hypoperfusion cause neurohormonal activation, inflammation and oxidative stress which, in turn, damage endothelial glycocalix and consequently impair endothelial function and fluid homeostasis, promote a prothrombotic and proapoptotic environment, thus leading to a vicious pathogenetic circle (25, 34). Inflammatory activation seem to persist beyond the acute event and may contribute to high rehospitalization rates of heart failure (35).

The acute phase protein hsCRP is produced by hepatocytes in response to inflammation and is the most commonly measured inflammatory biomarker because simple to assay and relatively stable in peripheral blood (36). In the Acute Study of Clinical Effectiveness of Nesiritide in Decompensated Heart Failure (ASCEND-HF) trial, a median hsCRP concentration of 12.6 mg/L was observed at hospital admission (Table 1), highlighting the exacerbation of an inflammatory state related to AHF (37). In the prospective analysis conducted by Matsumoto et al. to evaluate the association between CRP levels at hospital admission and long-term mortality in 527 patients with ADHF, the quartile with the lowest baseline hsCRP value was numerically the least representative (<0.3 mg/dl, *n* = 114) (38). In the prospective multicenter Acute decompensated heart failure syndrome (ATTEND) registry, an overall median hsCRP level at admission of 5.8 mg/L was observed, with the first tertile including patients with lowest hsCRP levels at baseline (<2.9 mg/L) as the least numerous subgroup (39). In a recent sub-analysis of the Epidemiology of Acute Heart Failure in the Emergency Departments (EAHFE) registry including 1,109 patients with CRP > 5 mg/L, the median CRP value was of 36.7 mg/L (40).

**Table 1:** Inflammatory biomarkers in acute heart failure.

Study (year)	Setting	Design	No. of patients	Baseline values of biomarkers	Key findings on the prognostic role of biomarkers
hsCRP					
ASCEND-HF sub-study (2014)	ADHF	Randomized trial	794	12.6 mg/L (IQR 5.23–30.5)	Persistently elevated or increasing hsCRP levels at day 30 after admission were predictive of 180-day mortality
ATTEND (2017)	AHF	Observational study	4777	5.8 mg/L (intertertile range 2.9–11.8)	Markedly elevated CRP levels (about >10 mg/L) at admission are associated with higher short-term cardiac and non-cardiac mortality
EAHFE registry sub-analysis (2022)	AHF	Observational study	1109	36.7 ± 49.1 mg/L	Short-term outcomes may be affected by corticosteroid therapy in patients with highly elevated signs of inflammation such as CRP > 40 mg/L
Matsumoto H et al. (2019)	ADHF	Observational study	527	132 patients with CRP ≥ 3.9 mg/dl	Admission CRP level was associated with an increased risk of long-term mortality
China PEACE 5p-HF Study (2023)	AHF	Observational study	1281	3.5 mg/l (1.5–9.7)	Patients with persistently high and very high trajectories of hsCRP had significantly higher mortality than those with the persistently low trajectory
IL-6					
BASEL-V study (2023)	AHF	Observational study	2042 acutely dyspneic subjects (1026 with a final diagnosis of AHF)	IL-6 higher in patients with AHF [11.2 (6.1–26.4) ng/L] vs. other causes of dyspnoea [9.0 (3.2–32.3) ng/L, *p* < 0.001]; IL-6 highest levels in cardiogenic shock [25.7 (14.0–164.2) ng/L] and lowest in patients with hypertensive HF [9.3 (4.8–21.6) ng/L]	IL-6 concentrations at hospital admission were a strong and independent predictor of long-term mortality
ASCEND-HF sub-study (2016)	ADHF	Randomized trial	883	14.1 pg/ml (IQR 8.1–26.4)	IL-6 levels were associated with higher all-cause mortality
PROTECT sub-study (2021)	AHF	Randomized trial	2033	11.1 pg/ml (IQR 6.6–20.9)	Temporal evolution patterns of IL-6 have additive prognostic value independent of changes in BNP
Suzuki et al. (2005)	ADHF	Observational study	73	84.7 ± 6.2 pg/ml	Peak IL-6 and percentage change of IL-6 between peak and 24 h correlated with pulmonary wedge pressure on admission and percentage change of pulmonary wedge pressure between peak and 24 h
IL-1β; sST2					
Pascal-Figual et al. (2019)	ADHF	Observational study	316	Median IL-1β 32.08 pg/ml (IQR 21.50–49.70) Median sST2 45.93 ng/ml (IQR 33.56–69.75)	IL-1β concentration at presentation was associated with prior HF hospitalizations, functional impairment, higher N-terminal pro–BNP and troponin T concentrations, and higher all-cause mortality; there was also a positive correlation between IL-1β and sST2.
PRIDE study (2007)	AHF	Observational study	599 acutely dyspneic subjects (209 with a final diagnosis of AHF)	sST2 concentrations higher in AHF patients (0.50 ng/ml, IQR 0.27–1.22) vs. others (0.15 ng/ml, IQR 0.06–0.42; *p* < 0.001)	Elevated sST2 concentrations strongly predicted death at 1 year among patients with AHF (hazard ratio 9.3; 95% CI 1.3–17.8), and percent change in sST2 concentrations was predictive of 90-day mortality independently from natriuretic peptide levels
Gal-3					
PRIDE study (2006)	AHF	Observational study	599 acutely dyspneic subjects (209 with a final diagnosis of AHF	Median Gal-3 concentrations higher AHF patients vs. others (9.2 vs. 6.9 pg/ml, *p* < 0.001).	Gal-3 levels were elevated in subjects with HF and associated with adverse outcomes over a 60-day period after presentation
Shah et al. (2010)	AHF	Observational study	115 patients from the overall PRIDE cohort (76 patients with diagnosis of AHF)	Median Gal-3 concentrations higher in AHF patients [15.0 ng/ml (11.1–19.7)] vs. others [11.0 ng/ml (9.1–14.4); *p* = 0.006]	The highest levels of Gal-3 were strongly associated with a higher risk of 4-year mortality, independent of LV dimensions, function, or RV pressures.Dyspneic patients with HF and Gal-3 levels above the median value had a 63% mortality; patients less than the median value had a 37% mortality (*p* = 0.003)
CA 125					
Kouris et al. (2003)	AHF	Observational study	77	22.4 U/ml (11.5–48.9)	Serum levels of CA125 were associated with HF severity and were independent predictive markers of re-hospitalizations
Miñana et al. (2020)	AHF	Observational study	2949	58.1 U/ml (25–129)	Elevated CA125 levels were strongly associated with the severity of congestion in AHF patients and specific factors, such as clinical parameters of congestion and tricuspid regurgitation severity, were significantly correlated with variations in CA125 concentrations
Llàcer et al. (2021)	AHF	Observational study	191	58 U/ml (23–129)	CA125 was positively and independently associated with the presence of peripheral oedema, pleural effusion and inferior vein cava dilation
GDF-15					
RELAX-AHF sub-study (2015)	AHF	Randomized trial	1106	Elevated baseline GDF-15 levels (>1200 ng/L) found in 99% of patients	Increasing GDF-15 concentrations predicted a higher risk of 60-day cardiovascular death or rehospitalization for HF or renal failure and 180-day cardiovascular death
MPO					
BASEL V study (2010)	AHF	Observational study	667 acutely dyspneic subjects (377 with a final diagnosis of AHF)	143 pmol/L (IQR 88–233)	MPO was an additive and independent predictor of mortality in AHF; MPO concentrations above the lowest tertile (>99 pmol/L) were associated with significantly increased 1-year mortality (hazard ratio 1.58, *p* = 0.02)

ADHF, acute decompensated heart failure; AHF, acute heart failure; BNP, B-type natriuretic peptide; CA 125, antigen carbohydrate 125; CI, confidence interval; CRP, C-reactive protein; Gal-3, galectin-3; GDF-15, Growth differentiation factor 1; HF, heart failure; hsCRP, high sensitivity C-reactive protein; IL-1, interleukin-1; IL-6, interleukin-6; IQR, interquartile range; IVC, inferior vena cava; MPO, myeloperoxidase; sST2, soluble suppression of tumorigenicity 2.

Moving to other biomarkers beyond CRP, some studies evaluated IL-6 and IL-1 as markers of inflammation. IL-6 promotes a shift from a neutrophilic to a mononuclear cell infiltrate and is the primary cytokine that mediates the transition from acute to chronic inflammation (41). It acts downstream of IL-1 and represents the primary stimulus for the liver production of CRP (42). IL-6 increases cardiomyocyte stiffness reducing titin phosphorylation (43). Additionally, IL-6 also impairs natriuresis (by stimulating ENaC in the distal renal tubule) and urinary IL-6 concentrations are associated with worsening renal function and diuretic resistance. IL-6, like TNF-alpha, can also produce myocardial dysfunction; IL-6 signaling plays a role in harmful effects in the myocardium as cardiac myocyte loss and contributes to the progression of compensatory LV hypertrophy to heart failure, trigger a series of pathological responses, such as oxidative stress, endothelial dysfunction, induction of myocyte apoptosis with adverse remodelling which ultimately leads to cardiomyocyte dysfunction (44). In a secondary analysis of the large prospective multicenter Basics in Acute Shortness of Breath EvaLuation (BASEL-V) study, a novel IL-6 immunoassay has been used to quantify systemic inflammation in AHF. The vast majority (84%) of patient with AHF had elevated IL-6 concentrations (*>*4.45 ng/L), with less than one third with other causes of dyspnea (45). In ASCEND-HF, the median baseline IL-6 value was 14.1 pg/ml (IQR 8.1–26.4 pg/ml) and 293 out of 883 patients recruited had baseline IL-6≥20.4 pg/ml (46). In a retrospective analysis of the PROTECT cohort the levels of both plasma IL-6 and BNP determined by high-sensitivity single molecule counting technology (Singulex Inc.). Patients with higher baseline IL-6 levels were older, had lower estimated glomerular filtration rates and higher BNP levels (47).

IL-1 is an apical inflammatory cytokine that is moderately elevated in most HF forms and is markedly elevated in ADHF, as measured by CRP and IL-6 that are surrogate biomarkers of IL-1 activity. Furthermore, sST2, encoded by gene ST2 located on human chromosome 2q12 (48), is a protein member of the IL-1 receptor family released under conditions of myocardial and vascular strain and plays an essential role in mediating myocardial remodeling and fibrosis (49). The ST2 protein is found both as a trans-membrane and a soluble form in serum. The trans-membrane form of ST2 plays a role in modulating responses of T helper type 2 cells, whereas the soluble form of ST2 is up-regulated in growth-stimulated fibroblasts. Despite the potential role played by ST2 in inflammation, significant parallels between ST2 and natriuretic peptides exist: the ST2 gene is markedly up-regulated in states of myocyte stretch, similar to the induction of the BNP gene (50).

To evaluate the efficacy of new therapies against the inflammatory IL-1 pathway such as anakinra, CRP (or hsCRP) and/or plasma IL-6 levels have been used as IL-1 surrogate biomarkers. In the Recently Decompensated Heart Failure Anakinra Response Trial (REDHART) left ventricular ejection fraction (LVEF) < 50% and CRP > 2 mg/L, a good correlation was observed between CRP levels at 12 weeks and changes in peak Vo_2_ (*R *= −0.57; *P *= 0.001) (51). In a small randomized pilot study conducted by Van Tassell et al. and evaluating the usefulness of anakinra in reducing systemic inflammation among 30 patients with ADHF and hsCRP ≥ 5 mg/L, hsCPR plasma levels were considered as a good marker to express IL-1 inflammatory pathway activation (52). Moreover a study conducted by Pascal-Figual et al. evaluated the relationship between IL-1β and sST2 in ADHF, as well as the prognostic role of elevated IL-1β and sST2 concentrations (53).

Taken together, these studies suggest that signs of systemic inflammation are frequently observed among patients with AHF, potentially with higher levels in patients with more severe clinical presentation (i.e., cardiogenic shock) as compared to those with less severe phenotypes.

Another important biomarker that correlate with inflammation and cardiac fibrosis with adverse remodeling is galectin-3, a soluble β-galactoside-binding protein secreted by activated macrophages, whose main action is to bind to and activate the fibroblasts that form collagen (54). Driven by the growing interest in this topic, new potential markers of inflammation have been related to AHF. Myeloperoxidase (MPO) is released from activated neutrophils, monocytes and endothelial cells in response to oxidative stress, especially after myocardial infarction, while growth differentiation factor 15 (GDF-15) regulates inflammatory and apoptotic pathways by inhibiting macrophage activation in a large number of pathological conditions, including AHF (55, 56).

Recently, carbohydrate antigen 125 (CA125), a large glycoprotein synthesized by mesothelial cells, has been validated as a reliable indicator of congestion and inflammation in patients with AHF.

Furthermore, another aspect deserving attention is the potential bidirectional relationship between HF decompensation and inflammation. A robust interconnection exists between inflammation, HF evolution and hemodynamic impairment (reduced cardiac output, elevated filling pressures) (57). This synergistic interplay assumes heightened significance during the acute stages of the disease. Recent studies demonstrated how conventional HF medications can exert a substantial “pleiotropic” effect in mitigating inflammation. The study of Mapelli et al. shows how sacubitril/valsartan have effects on HF biomarkers (in particular decreasing NT-proBNP and ST2 levels), indicating its ability to modulate the underlying pathophysiological processes associated with HF (58). The impact of sacubitril/valsartan has been also examined on circulating microRNAs (miRNAs) in patients with HF: miRNAs are small RNA molecules that play a crucial role in regulating gene expression and have been implicated in various cardiovascular diseases, including HF. The results of the study indicate that sacubitril/valsartan treatment leads to changes in the levels of circulating miRNAs that are known to be involved in the pathophysiological processes of HF, including also inflammation (59).

As demonstrated in the study of Campodonico et al., another drug used in AHF, levosimendan, has positive effects on inflammation: the results of the study demonstrate that acute hemodynamic improvement with levosimendan leads to changes in surfactant proteins in patients with advanced chronic HF. Specifically, there are alterations in the levels of certain surfactant proteins, indicating a potential impact on lung function. These findings suggest that the beneficial effects of levosimendan in HF may extend beyond the cardiovascular system and affect pulmonary function as well (60).

## Prognostic impact of inflammation in AHF

The prognostic role of inflammatory markers in both chronic and acute HF is well known (26, 61). Therefore, several studies have been conducted with the aim of evaluating whether the decreasing levels of these markers, in response to anti-inflammatory therapies, correlated with better outcomes.

The potential prognostic role and predictive value of hsCRP have been evaluated (62, 63). In patients with AHF, high levels of CRP at hospital admission have been associated with higher short-term cardiac and non-cardiac mortality, as demonstrated by the ASCEND-HF trial, the ATTEND study and the China Patient-centered Evaluative Assessment of Cardiac Events (PEACE 5p-HF) study (Table 1). In a biomarker study of the ASCEND-HF trial, higher baseline hsCRP levels were associated with longer hospital stay [0.57 days per log2 hsCRP in adjusted models; 95% confidence interval (CI) 0.33–0.81; *P *< 0.001], but were not associated with the composite short-term endpoint of death or worsening HF during index admission, 30-day death or HF readmission, or 180-day mortality. In both HFrEF and HFpEF subgroups, levels and changes of hsCRP were not differentially associated with outcomes. However, persistently elevated or increasing hsCRP levels at day 30 after admission were predictive of 180-day mortality (37).

In the ATTEND study, significant increases in all-cause, cardiac, and non-cardiac death were observed from the lowest to highest tertiles of CRP levels measured on admission (log-rank *P* < 0.001, *P* < 0.001, and *P* < 0.001, respectively). Among patients in the third, second and first tertile, the incidence rates of all-cause death was 34.1% (95% CI 31.5–36.7), 22.9% (95% CI 20.6–25.2) and 17.0% (95% CI 15.0–19.1), respectively. Furthermore, there were significant interactions of CRP levels with mortality when the patients were stratified by LVEF, with risk of cardiac mortality significantly greater in the subgroup of patients with reduced ejection fraction (40%) than in those with preserved ejection fraction (>40%, *P* = 0.047 for interaction) (39, 64). In PEACE 5p-HF, patients with persistently high and very high trajectories had a significantly higher risk of all-cause and cardiovascular mortality than those with the persistently low trajectory. Of note, CRP appears to have no role in the prognostic evaluation of AHF patients in the context of a concomitant infectious state. Non-infected patients with CRP > 12.3 mg/L had a doubled risk of death or readmission for HF, independent of other well-established predictors of prognosis. All-cause death or readmission for HF worsening occurred in 40% of the patients whose CRP value increased compared with 26.4% of those whose CRP decreased (*P *= 0.23). Evaluating the entire non infected sample, a lower CRP (<6.7 mg/L) was associated with better hospitalization-free survival, reinforcing the idea that stronger inflammatory activation carries a worse prognosis (65).

IL-6 elevations in both plasma and urine were associated with features of cardio-renal syndrome with decreased glomerular filtration rate, decreased diuretic responsiveness, and enhanced neurohormonal activation (66). Of note, in a recent analysis of BASEL V, IL-6 concentrations were a strong independent predictor of 1-year all-cause mortality among patients with AHF, in particular in patients without clinically overt infection at presentation. The observed 1-year all-cause mortality and HF rehospitalization rate were significantly higher in patients with elevated IL-6 levels compared to those with normal levels (*P* < 0.001 and *P *= 0.002, respectively). Furthermore IL-6 concentrations differed substantially among different AHF phenotypes with the highest concentrations observed in cardiogenic shock and the lowest in hypertensive HF or worsening HF, showing a correlation between the most severe AHF phenotype and a most extensive systemic inflammation. The addition of IL-6 also improved the predictive value of the BIOSTAT-CHF risk model for all-cause death (45). In an ASCEND-HF substudy, high baseline IL-6 values were associated with 30-day and 180-day mortality in patients with AHF. Moreover IL-6 levels closely correlated with NT-proBNP in this population (46). Furthermore, the temporal evolution patterns and the time-course of changes in the levels of IL-6 had additive prognostic value independent of BNP changes in patients with AHF, correlated with HF severity and predicted worsening HF (47, 67, 68). Finally, Miettinen et al. demonstred how IL-6 is linked to other pro-inflammatory cytokines, and elevated circulating levels of IL-6 and TNF-α were strongly associated with increased 1-year mortality in patients with ADHF, with poor outcome for ADHF patients with upper tertile levels of IL-6 and TNF-α (69).

In ADHF, IL-1β concentration at clinical presentation was associated with prior HF hospitalizations, higher NT-proBNP values and functional impairment. As continuous variables, IL-1β and sST2 concentrations were associated with higher all-cause mortality, with significantly higher IL-1β levels in patients who died during the first year after hospitalization (53). If the sST2 values currently do not have diagnostic role, they instead have a strong prognostic value in ADHF, as demonstrated by the PRIDE study: in a stratified analysis of only those patients with A HF and available ST2 results, ST2 > 0.20 ng/ml was an indipendent predictor of death, with a subsequently graded relationship between ST2 concentrations and mortality. Subjects above the ST2 median had 11-fold greater odds for death compared with those below the median (70).

Galectin-3 leads to progressive cardiac fibrosis, linking local inflammation and myocardial remodeling. It has been demonstrated that galectin-3 is a useful marker patients with AHF in predicting 60-day and long-term mortality. In an analysis conducted by van Kimmenade et al., an elevated level of galectin-3 was the best independent predictor of 60-day mortality or the combination of death/recurrent HF within 60 days, superior to NT-proBNP. Median concentrations of galectin-3 were significantly higher among those subjects dying by 60 days of follow-up than in those surviving (71).

V. Shah et al. have demonstrated a good correlation between galectin-3 concentrations and echocardiographic markers of ventricular diastolic and sistolic function. Furthermore, in dyspnoeic patients with ADHF, galectin-3 remained a significant predictor of 4-year mortality independent of echocardiographic markers of risk. Patients with galectin-3 levels above the median value had a 63% mortality; patients less than the median value had a 37% mortality (*P *= 0.003) (72).

Furthermore, high MPO and high GDF-15 concentrations were independently associated with worse outcomes in AHF, including higher mortality. MPO concentrations, despite their poor diagnostic accuracy for AHF, have shown an important prognostic role as indipendent predictors of 1-year mortality in AHF. In patients with AHF, higher mortality was observed when MPO concentrations at presentation were in the second (99–190 pmol/L, 37% mortality) and third (>190 pmol/L, 39% mortality) tertiles of MPO compared with the first tertile (≤99 pmol/L, 28% mortality, *P* = 0.058) (73). Results from the RELAX-AHF study showed that higher levels of GDF-15 at baseline and larger increases at day 2 and day 14 were associated with significantly increased risks of the combined endpoint of 60-day HF rehospitalizations or CV death (74).

In a multi-marker prognostic analysis in AHF, higher GDF-15 concentrations were associated with worse prognosis in AHF independently of BNP, in both patients with HFrEF and HFpEF. Patients discharged with both BNP and GDF-15 above the mean had a multivariate adjusted HR of 2-year death of 4.33 (95% CI 2.07–90.6, *P* < 0.001) when compared with the reference category (both BNP and GDF-15 below the mean) (75).

CA125, a complex glycoprotein encoded by the MUC16 gene in humans, has been recently studied in cardiovascular diseases, particularly in cases of decompensated HF and during the transition to clinical stability. Much evidence has recently supported the positive correlation of plasma levels of CA125 with congestion and inflammation. The reasons for elevated CA125 levels in decompensated HF are not well understood, but it appears that both hemodynamic and inflammatory factors play a role (31). One proposed mechanism is the activation of mesothelial cells in response to increased pressure, mechanical stress, and cytokine activation, leading to CA125 synthesis. Inflammatory cytokines like IL-1, TNF-α, and lipopolysaccharide have been shown to enhance CA125 secretion (76). In HF, there is evidence linking higher CA125 levels to congestion and inflammation which are strictly interconnected, and long-term venous congestion may trigger the inflammatory system, resulting in cytokine-driven CA125 synthesis and release by mesothelial cells, even when there is no visible serosal effusion (77).

As demonstrated in the study of Kouris et al.*,* elevated CA125 levels were significantly associated with the severity of congestion in HF patients (72). Around two-thirds of patients with AHF were found to have elevated CA125 levels. Another study by Miñana et al. investigated the factors influencing the plasma levels of CA125 in patients with AHF, identifying a strong association between elevated CA125 levels and the severity of congestion in AHF patients. Moreover, the authors identified specific factors, such as clinical parameters of congestion and the severity of tricuspid regurgitation, that were significantly correlated with variations in CA125 concentrations (73). Finally, Llàcer et al. compared the effectiveness of two biomarkers, CA125 and NT-proBNP, in evaluating congestion in patients with AHF, showing that both CA125 and NT-proBNP were useful indicators of congestion in AHF, with elevated levels of both biomarkers being associated with increased severity of congestion (74). Of note, CA125 exhibited a stronger correlation with congestion compared to NT-proBNP. The study also found that the combination of these biomarkers improved the accuracy of evaluating congestion levels in AHF patients, allowing for a more precise and reliable diagnosis and potentially aiding in better treatment decisions. These findings suggest that CA125 could be used as a valuable biomarker for assessing the severity and prognosis of AHF, providing clinicians with useful information for patient management and treatment strategies.

**Figure 1:**
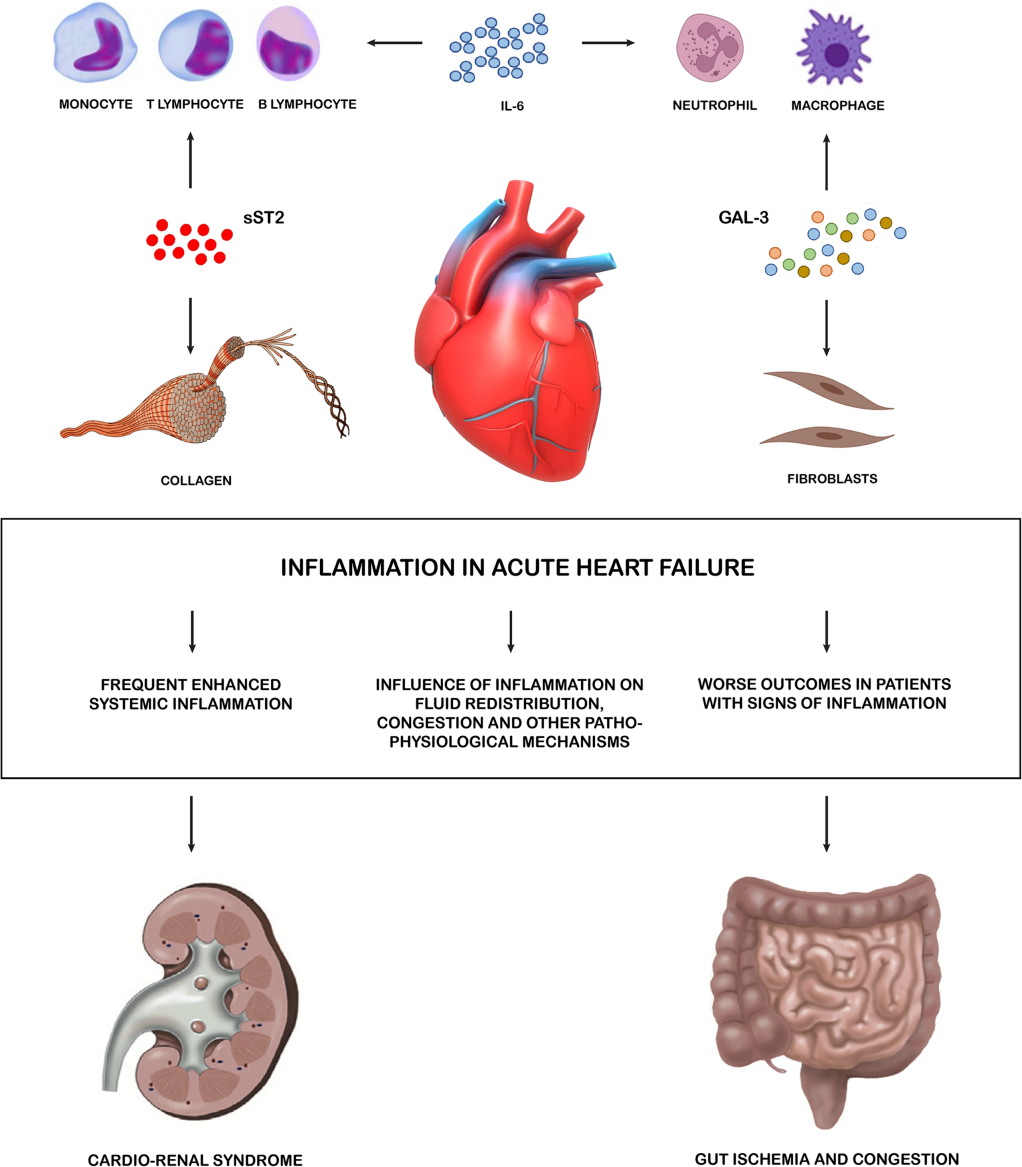
Inflammation in acute heart failure. Systemic inflammation has been proved to be frequently enhanced during AHF, with high levels of different inflammatory biomarkers identified in relevant subset of patients. Such biomarkers have also been involved in key pathophysiological pathways leading to HF decompensation. These pro-inflammatory cytokines allow the crosstalk between the heart and multiple peripheral organ systems. The main effectors of inflammations are the lymphatic tissue cells which, through cytokines, also activate fibroblasts and lead to the formation of collagen. Kidney and gut are targets of these negative effects. sST2, soluble suppression of tumorigenesis-2; GAL 3, galectin 3; AHF, acute heart failure.

Therefore, available evidence suggests that different biomarkers involved in inflammatory pathways (CRP, IL-6, IL-1 axis, Galectin-3, MPO, GDF-15 and CA-125) have a strong prognostic impact in patients with AHF, and their increased circulating levels secondary to enhanced systemic inflammation are associated with worse outcomes and may represent interesting therapeutic targets (Figure 1).

## Anti-inflammatory therapies in AHF

Over the past decades, several anti-inflammatory therapies have been investigated for their potential efficacy in treating HF, including colchicine, methotrexate, xanthine oxidase inhibitors, monoclonal antibodies targeting inflammatory cytokines, and corticosteroids. However, the majority of these studies have focused on the role of anti-inflammatory drugs in chronic HF, while evidence is scarce about their use in patients with AHF (Table 2). Different immune mechanisms might be involved in the acute and chronic phases of HF. Innate immune responses might be more relevant targets in *de novo* HF, whereas adaptive immune responses might be more prominent in chronic or acutely decompensated HF settings (78).

**Table 2:** Studies evaluating anti-inflammatory therapies in heart failure.

Study (year)	Design	No. of Patients	Setting	Type of intervention and comparator	Endpoints	Key results
Van Tassell et al. (2016)	Randomized, double-blinded, placebo-controlled pilot study	30	ADHF, EF < 40%, and CRP levels ≥ 5 mg/L	Anakinra 100 mg twice daily for 3 days followed by once daily for 11 days or matching placebo	Reduction of systemic inflammatory response comparing the AUC at 72 h for hsCRP between allocation groups	Significant reduction of systemic inflammatory response with IL-1 blockade
REDHART (2017)	Single-center, prospective, double-blinded, placebo-controlled study	60	Acute systolic HF (EF < 50%) with hsCRP >2 mg/L	Subcutaneous injections with anakinra 100 mg for 2 weeks, 12 weeks, or placebo	Improvement of peak aerobic exercise capacity (measurement of peak oxygen consumption and ventilatory efficiency)	No change in peak oxygen consumption at 2 weeks, improvement with continued anakinra for 12 weeks
Zhang et al. (2008)	Prospective, single-arm, non placebo-controlled, observational study	35	Decompensated congestive HF refractory to conventional therapy	Prednisone (1 mg/kg/ day with maximum dosage of 60 mg/day) added to the standard treatment	Daily urine volume, assessed dyspnea, global clinical status, changes in renal function.	Induction of potent diuresis, relief of congestive symptoms and improvements in clinical status and renal function
COPE-ADHF (2014)	Multicenter, non-blinded, randomized trial	102	ADHF	1 dose of dexamethasone (20 mg/day) intravenously followed by prednisone (orally, daily, 1 mg/kg/day) for 7 days	CV death within 30 days; patient-assessed dyspnea, change from baseline in serum creatinine levels and physician-assessed global clinical status at day 7	Significant creatinine reduction after 7 days of treatment, CV death reduction at 30 days, improvement in patient-assessed dyspnea and physician-assessed global clinical status
Parissis et al. (2004)	Randomized, placebo-controlled trial	27	Decompensated advanced HF	10 min intravenous levosimendan bolus of 6 ug/kg followed by continuous infusion	Reduction of circulating pro-inflammatory cytokines and soluble apoptosis mediators in patients with decompensated severe HF	Significant reduction of circulating pro-inflammatory cytokine IL-6, TNF-alfa and soluble apoptosis mediators, such as soluble Fas and Fas ligand

ADHF, acute decompensated heart failure; AUC, area under the curve; CV, cardiovascular; EF, ejection fraction; HF, heart failure; hsCRP, high sensitivity C reactive protein; HHF, hospitalization for heart failure.

To date, only a few studies evaluated the effect of anti-inflammatory therapy in the setting of AHF. Monoclonal antibodies targeting inflammatory cytokines, such as canakinumab and anakinra, have shown promising results in reducing inflammation yet not definitive results in improving outcomes among patients with AHF. A pilot randomized trial enrolled 30 patients with ADHF, LVEF < 40% and elevated hsCRP levels (≥5 mg/L) that were randomized 1:1 to either anakinra 100 mg twice daily for 3 days followed by once daily for 11 days or matching placebo. Anakinra reduced CRP at 72 h by 61% versus baseline, compared with a 6% reduction among patients receiving placebo (*p* = 0.004). The study was designed to evaluated only changes in inflammatory markers and not functional or clinical outcomes (52). In the REDHART trial, 60 patients with LVEF <50%, recent hospitalization for decompensated HF and elevated CRP levels (>2 mg/L), within 14 days of hospital discharge, were randomized 1:1:1 to daily subcutaneous injections with anakinra 100 mg for 2 weeks, 12 weeks, or placebo. The aim of the study was to evaluate whether IL-1 blockade with anakinra would improve aerobic exercise capacity in patients with recently decompensated systolic HF. The evidence of enhanced systemic inflammation, in particular of the IL-1 inflammatory pathway, was demonstrated by elevated CRP plasma levels (>2 mg/L, measured by high-sensitivity assay). CRP at 12 weeks was reduced by a median of 66% in the 12-week anakinra group (*P *= 0.011), whereas by 12 weeks the CRP levels in the anakinra 2-week group were no longer significantly different from baseline, with a good correlation between CRP levels at 12 weeks and changes in peak Vo_2_ (*R *= −0.57; *P *= 0.001). No change in peak VO2 occurred at 2 weeks in patients treated with anakinra, although an improvement was seen in those who continued anakinra for 12 weeks (51).

Is well known that colchicine may reduce myocardial stiffness and cardiac hypertrophy also through various anti-inflammatory actions, such as inhibiting the activation of the nucleotide-binding domain-like receptor protein 3 inflammasome, which leads to the maturation of IL-1β and IL-18. It also hampers the movement of neutrophils by blocking tubulin polymerization and microtubule formation, reduces IL-1 production in activated neutrophils, and decreases the expression of TNF-α receptors in macrophages and endothelial cells (79). In a retrospective study evaluating the role of colchicine to treat acute gout flares in patients with ADHF, colchicine use was associated with a lower rate of in-hospital all-cause and cardiovascular mortality, but patients who received colchicine had longer hospital stays. There was no significant difference in 30-day readmission rates between patients receiving and not receiving colchicine (80). Regarding steroid therapy in AHF, some trials have been conducted evaluating its inflammatory role with discordant results so far. A single-arm, observational study by Zhang et al. investigated the potential benefit of adding prednisone to usual care treatment in patients with refractory decompensated congestive HF. The study enrolled 35 patients with refractory decompensated HF who received prednisone in addition to usual care. The primary endpoints of the study were the effects on daily urine volume, patient- and physician-assessed dyspnoea and global clinical status, and changes in renal function. The results of the study showed that treatment with prednisone, on top of usual care, induced potent diuresis and was associated with an improvement in congestive symptoms, clinical status and renal function (81). In the Cardiac Outcome Prevention Effectiveness of Glucocorticoids in Acute Decompensated Heart Failure (COPE-ADHF) trial, 102 patients with ADHF were randomized to receive corticosteroids (single dose of 20-mg dexamethasone followed by 1 mg/kg prednisone daily with a maximum dose of 60 mg daily for 7 days) plus diuretic therapy or standard diuretic therapy alone. The primary endpoint of the study was a change from baseline in serum creatinine at day 7 and cardiovascular death within 30 days. There was a remarkable reduction in creatinine after 7 days of treatment and a reduction in 30-day cardiovascular death in the glucocorticoid group (82). Recently, the large observational CORTicosterioids in Acute Heart Failure (CORT-AHF) study confirmed the safety of new onset of systemic corticosteroids in AHF, reporting no evidence of harm among 11,356 patients with AHF enrolled in EAHFE registry. This was confirmed both in patients with or without concomitant chronic obstructive pulmonary disease (COPD) (83).

Finally, levosimendan was associated to a significant decrease in circulating pro-inflammatory cytokines and also a reduction in soluble apoptosis mediators demonstrating that this medication has a positive effect on reducing inflammation and cell death processes in patients with decompensated advanced HF (84).

Therefore, although preliminary evidence suggests the safety of anti-inflammatory therapy in the AHF setting, especially for systemic corticosteroids, no definitive proof of efficacy has been demonstrated in patients with AHF and concomitant systemic inflammation.

## Ongoing studies and future perspectives

Some studies testing anti-inflammatory therapy in AHF are ongoing. Beyond the randomized Dexamethasone Versus Prednisone in Heart Failure Patients, Hospitalized With Exacerbation of Chronic Obstructive Pulmonary Disease trial (NCT02237820), that is focused on HF patients hospitalized with exacerbation of COPD, other studies are specifically focusing on the impact on anti-inflammatory strategies in AHF.

The Recently Decompensated Heart failure Anakinra Response 2 Trial (REDHART2) planned to enrol 102 patients with recently decompensated HF (before hospital discharge), LVEF ≤ 40% and hsCRP >2 mg/L, that are randomized 2:1 to anakinra 100 mg for 24 weeks or placebo (NCT03797001). The primary objective is to determine whether sustained anakinra treatment determines an improvement in aerobic exercise capacity, with peak VO2 change at 24 weeks as primary endpoint.

The ongoing Randomized Double-blind Trial to Study the Benefit of Colchicine in Patients With Acutely Decompensated Heart Failure (COLICA) study is randomizing 278 patients with ADHF and either reduced or preserved LVEF to colchicine 0.5 mg or placebo, initiated within the first 24 h of hospitalisation and administered for 8 weeks (NCT04705987). The primary objective of the study is the reduction of NT-proBNP after 2 months of treatment. Of note, evidence of systemic inflammation is not needed to be enrolled in the COLICA trial.

The Effect of Short-Term Prednisone Therapy on C-Reactive Protein Change in Emergency Department Patients With Acute Heart Failure and Elevated Inflammatory Marker (CORTAHF) trial is including 120 patients with AHF evaluated at the ED, randomized 1:1 to prednisone 40 mg for 7 days plus standard therapy or standard therapy alone (NCT05668676). Of note, prednisone therapy is initiated in the ED and continued up to 7 days. The primary endpoint is change in CRP from baseline to day 7, and other clinical and functional outcomes will be evaluated as secondary endpoints.

The multicenter, randomized, open-label, controlled study to evaluate the efficacy and safety of corticoSTEROids added to standard therapy in patients with Acute Heart Failure (STERO-AHF) pilot trial will enrol 120 patients hospitalized for AHF, irrespective of LVEF, with documented diuretic resistance (according to current guidelines) (5) and with evidence of sustained systemic inflammation (CRP ≥ 20 mg/L) (NCT05809011). Patients will be randomized 1:1 to or standard-of-care plus corticosteroid therapy for up to 7 days (single-bolus intravenous dexamethasone 20 mg on day 1 followed by oral prednisone 1 mg/kg daily—maximum 60 mg daily—from day 2 to day 7) or standard-of-care alone. The two primary endpoints are diuretic response, defined as absolute body weight change from baseline to day 8 (or to discharge or to the occurrence of death) per 40 mg total dose of administered intravenous furosemide or equivalent, and early clinical benefit, defined as a hierarchical composite of all-cause death, worsening HF, or change in patient-reported dyspnea (quantified by the visual analogue scale) from baseline to day 8 (or to discharge or to the occurrence of death).

Hopefully, these ongoing studies will help defining the role of pharmacological or interventional anti-inflammatory therapies in patients with AHF episodes.

## Conclusions

Different inflammatory pathways can be implicated in different HF stages, also including the specific setting of AHF. Inflammation can be both a cause and consequence of HF decompensation and seems to play a pathogenetic and prognostic role, with several inflammatory mediators and cytokines up-regulated and associated with worse prognosis in patients with AHF. Despite numerous studies validating the association between specific inflammatory biomarkers and AHF, results from clinical trials have proven conflicting results, with some recent trials showing encouraging preliminary findings, but without definitive and convincing evidence supporting the use of anti-inflammatory therapies in this setting, yet. Managing inflammation is still a clinical challenge in AHF, and ongoing studies will help defining the role of different anti-inflammatory pharmacological approaches (corticosteroids, colchicine, anti-IL-1 therapy) in improving AHF outcomes.
